# Alpha-Synuclein Preserves Mitochondrial Fusion and Function in Neuronal Cells

**DOI:** 10.1155/2019/4246350

**Published:** 2019-11-23

**Authors:** Gaia Faustini, Elena Marchesan, Laura Zonta, Federica Bono, Emanuela Bottani, Francesca Longhena, Elena Ziviani, Alessandra Valerio, Arianna Bellucci

**Affiliations:** ^1^Department of Molecular and Translational Medicine, University of Brescia, Viale Europa, 11, 25123 Brescia, Italy; ^2^Department of Biology, University of Padova, Via Ugo Bassi, 58b, 35131 Padova, Italy; ^3^Laboratory of Preventive and Personalized Medicine, University of Brescia, Viale Europa, 11, 25123 Brescia, Italy

## Abstract

Dysregulations of mitochondria with alterations in trafficking and morphology of these organelles have been related to Parkinson's disease (PD), a neurodegenerative disorder characterized by brain accumulation of Lewy bodies (LB), intraneuronal inclusions mainly composed of *α*-synuclein (*α*-syn) fibrils. Experimental evidence supports that *α*-syn pathological aggregation can negatively impinge on mitochondrial functions suggesting that this protein may be crucially involved in the control of mitochondrial homeostasis. The aim of this study was to assay this hypothesis by analyzing mitochondrial function and morphology in primary cortical neurons from C57BL/6JOlaHsd *α*-syn null and C57BL/6J wild-type (wt) mice. Primary cortical neurons from mice lacking *α*-syn showed decreased respiration capacity measured with a Seahorse XFe24 Extracellular Flux Analyzer. In addition, morphological Airyscan superresolution microscopy showed the presence of fragmented mitochondria while real-time PCR and western blot confirmed altered expression of proteins involved in mitochondrial shape modifications in the primary cortical neurons of *α*-syn null mice. Transmission electron microscopy (TEM) studies showed that *α*-syn null neurons exhibited impaired mitochondria-endoplasmic reticulum (ER) physical interaction. Specifically, we identified a decreased number of mitochondria-ER contacts (MERCs) paralleled by a significant increase in ER-mitochondria distance (i.e., MERC length). These findings support that *α*-syn physiologically preserves mitochondrial functions and homeostasis. Studying *α*-syn/mitochondria interplay in health and disease is thus pivotal for understanding their involvement in PD and other LB disorders.

## 1. Introduction

The pathological aggregation of *α*-synuclein (*α*-syn) and its deposition in proteinaceous inclusions named Lewy bodies (LB) is the key pathological hallmark of LB disorders such as Parkinson's disease (PD). This is the most common neurodegenerative movement syndrome and is characterized by a progressive loss of dopaminergic neurons of the nigrostriatal system.

The aggregation of pathological *α*-syn is thought to be the major agent of PD pathophysiology [1], but mitochondrial deficits have been largely described as crucial pathogenic events in the pathogenesis of PD [2]. Indeed, a bidirectional interplay between *α*-syn and mitochondrial dysfunction has been described, since *α*-syn aggregates may negatively impinge on mitochondrial homeostasis and dynamics, while mitochondrial dysfunctions severely affect *α*-syn deposition [3].

It has been reported that a fraction of soluble *α*-syn directly interacts with mitochondria-associated endoplasmic reticulum (ER) membranes (MAM) [4], influencing mitochondrial fusion and fission. Interestingly, *α*-syn aggregation produces mitochondrial fragmentation or mitochondrial respiration failure and death in cell-based models of PD [5–7]. Furthermore, mitochondrial protein import and protein degradation may impact on *α*-syn and mitochondrial physiological functions, but their reciprocal modulation is still to be elucidated [8]. On the other hand, a protective role of *α*-syn was identified in neurons exposed to oxidative stress [9], while the effect of several neurotoxins on mitochondria deficiency is thought to be mediated, at least in part, by *α*-syn aggregation [3, 10]. For instance, elevated levels of *α*-syn promote the toxic action of 1-methyl-4-phenyl-1,2,3,6-tetrahydropyridine (MPTP), which inhibits mitochondrial complex I [11], that is deficient in PD brains [12, 13]. Contrariwise, *α*-syn-deficient mice exposed to rotenone exhibited a pronounced degeneration of dopamine neurons exceeding that observed in wild type (wt) mice [14], even if silencing *α*-syn was reported to prevent neuron degeneration in an *in vitro* model [15]. These findings fit with the hypothesis that alterations of *α*-syn may contribute to bioenergetics defects inducing mitochondrial dysfunctions and PD onset. This notwithstanding, *α*-syn deficiency *per se* may also negatively impinge on mitochondrial homeostasis [16], supporting that the metabolism and function of healthy neurons may depend on the expression levels and conformation of *α*-syn and on the critical interplay between *α*-syn and mitochondria, with imbalances in their reciprocal modulation leading to neuronal impairment [3].

The aim of this study was to assay the relevance of *α*-syn function on mitochondrial homeostasis by assessing whether primary cortical neurons, produced from C57BL/6JOlaHsd mice carrying a spontaneous deletion of *α*-syn locus (*α*-syn null) [17], may present alterations of mitochondrial function and morphology when compared to those of C57BL/6J wt animals.

The Oxygen Consumption Rate (OCR) of cortical neurons from wt or *α*-syn null mice was evaluated by a Seahorse-based analysis both in basal condition and after exposure to the Complex I inhibitor rotenone. Mitochondria morphology was studied by using both Airyscan superresolution microscopy on mito-YFP-transfected neurons and transmission electron microscopy (TEM). Finally, the expression of proteins mediating mitochondria fusion and fission was also evaluated by real-time polymerase chain reaction (PCR) and western blotting (WB). These studies revealed a reduced mitochondrial respiration of *α*-syn null neurons and an increased susceptibility to rotenone administration. These cells also exhibited an increased mitochondrial fragmentation and alterations in the key proteins mediating mitochondrial fission and fusion, such as mitofusin (Mfn1) and the dynamin-like GTPase Opa1. Finally, TEM studies showed that the primary cortical neurons from *α*-syn null mice exhibited a decreased number of mitochondria-ER contacts (MERCs) and a significant increase in ER-mitochondria distance.

Our findings strongly support that, by orchestrating mitochondrial fusion and functions and preserving MERCs, *α*-syn physiologically acts as a determining regulator of mitochondrial homeostasis.

## 2. Materials and Methods

### 2.1. Animals

C57BL/6J wt (Charles River, Wilmington, MA) and C57BL/6JOlaHsd (Harlan Olac Bicester, UK) mice were bred in our animal house facility at the Department of Molecular and Translational Medicine of University of Brescia, Brescia, Italy. Animals were maintained under a 12 h light-dark cycle at a room temperature (rt) of 22°C and had ad libitum food and water. All experiments were made in accordance to Directive 2010/63/EU of the European Parliament and of the Council of 22 September 2010 on the protection of animals used. All experimental and surgical procedures for the preparation of primary cortical neuronal cell cultures from 18-day embryos were conformed to the National Research Guide for the Care and Use of Laboratory Animals and were approved by the Animal Research Committees of the University of Brescia (Protocol Permit 719/2015-PR). All achievements were made to minimize animal suffering and to reduce the number of animals used.

### 2.2. Primary Cortical Neurons

Primary cortical neurons were dissected from C57BL/6J wt control mice and C57BL/6JOlaHsd *α*-syn null mice, carrying a spontaneous deletion of the *α*-syn locus [17], at embryonic day 18 according to previously described protocols [18]. After dissociation with Accumax (Sigma-Aldrich, Milan, Italy), the single cells were resuspended in a Neurobasal medium (Thermo Fisher Scientific, Massachusetts, USA) containing 100 *μ*g/ml penicillin, 100 *μ*g/ml streptomycin (Sigma-Aldrich, Milan, Italy), 2 mM glutamine (EuroClone, Milan, Italy), and 1% B27 supplement (Thermo Fisher Scientific) and then centrifuged. Cell counts and viability assays were performed using the Trypan Blue exclusion test. Neurons were seeded onto glass coverslides in 24-well plates for imaging analyses, Seahorse XFe24-well plates for energetic analyses, 24-well plates for TEM analyses or Petri dishes coated with 10-12 *μ*g/ml poly-D-lysine for quantitative analyses. Cells were maintained at 37°C under a humidified atmosphere of 5% CO_2_ in the Neurobasal medium for 8-10 days *in vitro* (DIV).

### 2.3. Cortical Neuron Respirometry Analysis

Seahorse XF Cell Culture Microplates (Seahorse Biosciences, Agilent Technologies, USA) were used to seed 45,000 cortical neurons per well. At day 8, cells were treated with 100 nM rotenone, which was directly added in the cell culture media for 1 h and analyzed the following day. The medium was replaced with freshly prepared Seahorse XF Base Media (25 mM glucose, 0.25 mM sodium pyruvate, and 1 mM L-glutamine, pH 7.4) in a non-CO_2_ incubator for 1 h prior to the assay, then loaded on a Seahorse XF24 Extracellular Flux Analyzer (Seahorse Biosciences). The XF Cell Mito Stress Test (Agilent technologies) was performed, after 3 cycles of basal condition, sequentially injecting 1 *μ*M oligomycin, 0.5 *μ*M carbonyl cyanide-4-(trifluoromethoxy)phenylhydrazone (FCCP), and 0.5 *μ*M rotenone plus 0.5 *μ*M antimycin A. Oxygen Consumption Rate and Extracellular Acidification Rate (ECAR) were measured every three cycles of 3 min mix after the injection.

Normalization was performed by using the Bio-Rad DC™ protein assay kit (Bio-Rad Laboratories, California, USA).

Basal respiration was measured as the last rate before the first injection minus the nonmitochondrial respiration rate deriving from the rotenone/antimycin injection. Maximal respiration was considered the maximum rate measurement after the FCCP injection minus the nonmitochondrial respiration rate. The ATP-linked respiration was calculated as the subtraction of the last measurement before oligomycin injection and the minimum rate after oligomycin injection.

### 2.4. Mitochondrial Isolation and Seahorse Analysis

Liver, cortex, and midbrain tissues were explanted from two-month-old mice after cervical dislocation. After five washes with MIB1 buffer (210 mM D-mannitol, 70 mM sucrose, 5 mM HEPES, 1 mM EGTA and 0.5% free fatty acid BSA), tissues were homogenate and centrifuged at 600g for 10 min at 4°C. The supernatant was then centrifuged at 7000g for 10 min at 4°C. After washing the pellet with MIB1 by centrifugation at 7000g for 10 min at 4°C, the pellet was resuspended in MAS1 (220 mM D-mannitol, 70 mM sucrose, 2 mM HEPES, 1 mM EGTA, 0.2% free fatty acid BSA, 10 mM KH_2_PO_4_, 5 mM MgCl_2_, 10 mM glutamate, 5 mM malate, and 10 mM succinate, pH 7.2).

The total mitochondrial extract was quantified by using the Bio-Rad DC™ protein assay kit and 7 *μ*g per well was loaded in the 24-well Seahorse plate and centrifuged at 600g for 20 min at 4°C.

The XF Cell Mito Stress Test was performed, after one cycle of basal condition, sequentially injecting 4 mM ADP, 2.5 *μ*g/ml oligomycin, 4 *μ*M FCCP, and 4 *μ*M rotenone plus 4 *μ*M antimycin A.

Basal respiration was measured as the first basal rate minus the nonmitochondrial respiration rate deriving from the rotenone/antimycin injection. State III was calculated as the measurement after the ATP injection minus the nonmitochondrial respiration rate, State IV_0_ as the measurement after oligomycin minus the last rate, and State III_u_ as the measurement after FCCP injection minus the last rate.

### 2.5. Mito-Yellow Fluorescent Protein (YFP) Transfection and Immunofluorescence Staining

For imaging analysis, 80,000 primary cortical neurons were seeded onto poly-D-lysine-coated glass coverslides in 24-well plates. At 8 days of differentiation, neurons were transfected with pEYFP-Mito (Catalog #6115-1, Clontech) by using Lipofectamine 3000 (Life Technologies, California, USA), according to the manufacturer's instructions.

Fixed neurons were permeabilized in PBS 0.1 M supplemented with 20% methanol and 0.1% Triton X-100, incubated for 1 h at rt in blocking solution (2% Normal Goat Serum (NGS), 3% Bovine Serum Albumin (BSA), and 0.1% Triton-X100 in PBS 0.1 M), and then with the primary antibody (Microtubule-Associated Protein 2 (MAP-2), Merck Millipore, Burlington, Massachusetts, USA) in blocking solution overnight at 4°C. Neurons were washed with 0.1% Triton X-100 PBS 0.1 M and incubated with the fluorochrome-conjugated secondary antibody (goat anti-mouse cy3, Jackson ImmunoResearch, Cambridge, UK) in 0.1% Triton X-100 PBS 0.1 M plus 1 mg/ml BSA for 1 h at rt. After three washes in 0.1% Triton X-100 PBS, cells were mounted onto superfrost slides using a VECTASHIELD mounting medium for fluorescence (Vector Laboratories, Burlingame, CA) and observed by means of a Zeiss confocal laser microscope LSM 880 (Carl Zeiss, Oberkochen, Germany) with Airyscan superresolution and *z*-stack with the height of the sections scanning≅1 *μ*m. Images (1024 × 1024 pixels) were then reconstructed using Zen lite 2.3 (Carl Zeiss).

### 2.6. Quantification of Mitochondrial Morphology

The number of mitochondria and the total area of mitochondria per neuron were analyzed by using the macro of ImageJ Software designed by Dagda et al. [19] with minor modification to differentially analyze cell bodies and dendrites. All the *z*-stack images were processed to maximum intensity projection. The acquisition parameters during confocal imaging were maintained constant for all the image settings used for the analysis.

The interconnectivity between mitochondria was analyzed using the mitochondrial network analysis (MiNA) toolset [20].

### 2.7. Real-Time PCR

Total RNA was extracted from wt and *α*-syn null cortical neurons using an RNA extraction kit (RNeasy Mini Kit, Qiagen, Hilden, GE) according to the manufacturer's recommendations. Two micrograms of RNA was retrotranscribed by using a QuantiTect Reverse Transcription Kit (Qiagen) according to the manufacturer's instructions. Real-time PCR was performed by using a SYBR Green Master Mix (Applied Biosystems, Foster City, USA) and the following primer pairs: Mfn1 for *ACAAGCTTGCTGTCATTGGG* Mfn1 rev *TCGACACTCAGGAAGCAGTT*; Mfn2 for *ATATAGAGGAAGGTCTGGGCCG*, Mfn2 rev *CCGCATAGATACAGGAAGAAGGG*; Opa1 rev *GTCATTGTCGGAGCAGGAATC*, Opa1 for *TTCACTAAGGATTGGCAGACTT*; and GAPDH for *TCAACAGCAACTCCACTCTT*, GAPDH rev *CCAGGGTTTCTTACTTACTCCTTGG.*

The ViiA7 Real-Time PCR system (Life Technologies, Grand Island, NY, USA) was used for 40 cycles of 95°C for 15 s and 60°C for 1 min. mRNA expression was normalized to glyceraldehyde 3-phosphate dehydrogenase (GAPDH) gene expression.

### 2.8. Western Blot Analysis

Total proteins were extracted with a Radioimmunoprecipitation Assay (RIPA) buffer made up with 50 mM Tris-HCl pH 7.4, 150 mM NaCl, NP-40 1%, sodium deoxycholate 0.1%, sodium dodecyl sulfate (SDS) 0.1%, 1 mM NaF, and 1 mM NaVO_4_ plus complete protease inhibitor mixture (Roche Diagnostics, Mannheim, Germany). Protein concentration in the samples was measured by using the Bio-Rad protein assay kit. Equal amounts of proteins (30 *μ*g) were run on 10% polyacrylamide gels and transferred onto polyvinylidene fluoride (PVDF) membrane. Densitometric analysis of the bands was performed by using ImageJ software and all bands were normalized to Tom20 levels as a control of equal loading of samples in the total protein extracts. For densitometry analysis of bands, each experimental condition was performed in quadruplicate and the resulting data were subjected to statistical analysis. The primary antibodies used for western blot analysis were the following: Opa1 (1 : 1000; Abcam; ab42364), Mfn2 (1 : 1000; Abnova; H00009927-M03) and Tom20 (1 : 1000; Santa Cruz; sc-11415). Secondary antibodies used were sheep anti-mouse or donkey anti-rabbit HRP (GE Healthcare, Chicago, USA).

### 2.9. Transmission Electron Microscopy Ultrastructural Morphological Analysis

For ultrastructural morphological analysis, cortical neurons were fixed with Immunofix (BioOptica, Milan, Italy) for 1 hour. After rinsing in 0.1 M cacodylate buffer with 1% tannic acid, samples were postfixed in 1 : 1 2% OsO4 and 0.2 M cacodylate buffer for 1 h. Samples were rinsed, dehydrated in ethanol and embedded in Epon resin. Ultrathin sections were imaged on a Tecnai-20 electron microscope (Philips-FEI).

### 2.10. Analysis of TEM Mitochondria-ER Contacts

The number of mitochondria-ER contacts has been analysed for ultrastructure in a minimum of 250 contacts per condition. Morphometric measurements were carried out using ImageJ. For calculations of the mitochondria-ER distance, *n* > 5 mitochondria per image in 60 images per condition were considered and a minimum distance of ER located in a 40 or 10 nm radius from the considered mitochondria was computed.

## 3. Results and Discussion

The aggregation of *α*-syn has been repeatedly associated with mitochondrial dysfunctions [16], but the physiological role of *α*-syn in mitochondrial function still needs to be elucidated in order to achieve extensive comprehension of *α*-syn/mitochondria interplay.

To probe whether *α*-syn can physiologically influence mitochondrial function, we examined both mitochondrial respiration and morphology in primary cortical neurons derived from C57BL/6J wt and C57BL6JOlaHsd *α*-syn null mice.

**Figure 1:**
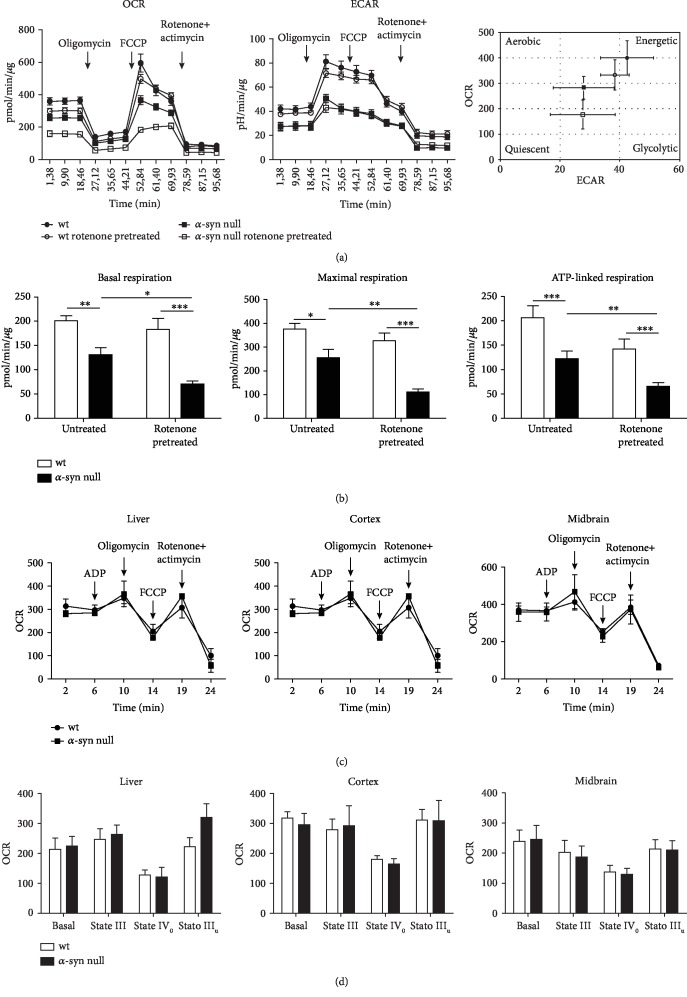
Seahorse-based mitochondrial respiration assay on primary cortical neurons or mitochondria purified from wt or *α*-syn null mice. (a) The two graphs show time-dependent changes in OCR and ECAR measured sequentially injecting oligomycin, FCCP, and rotenone plus antimycin A on primary cortical neurons of wt and *α*-syn null mice in basal conditions or after rotenone exposure. Basal OCR and ECAR values (before oligomycin injection) were plotted in the energy map to illustrate the difference in the cell metabolic profile. (b) Basal respiration (first injection minus the rotenone/antimycin injection), maximal respiration (rate after the FCCP injection minus the nonmitochondrial respiration rate), and ATP production (measurement before oligomycin injection and after the oligomycin injection) showed a decreased respiration of *α*-syn null cortical neurons that are significantly affected by rotenone treatment. (c) OCR of mitochondria purified from the liver, cortices, and midbrains was measured sequentially injecting ADP, oligomycin, FCCP, and rotenone plus antimycin A. Note the absence of changes between C57BL/6J wt and C57BL/6JOlaHsd *α*-syn null mice. (d) The basal respiration, the State III, the State IV_0_, and the State III_u_ did not show differences in the OCR of liver, cortex, and midbrain extracts between wt and *α*-syn null mice. ^∗^*P* < 0.05, ^∗∗^*P* < 0.01, and ^∗∗∗^*P* < 0.001, two-way ANOVA+Bonferroni's postcomparison test. Data are presented as mean ± standard error of the mean (SEM) (*n* = 12).

In particular, mitochondrial respiration of murine primary cortical neurons of wt and *α*-syn null mice was analyzed by using the Seahorse XFe24 Extracellular Flux Analyzer measuring the OCR and the ECAR (Figure 1(a)). The ATP synthase inhibitor oligomycin, the mitochondrial oxidative phosphorylation uncoupler FCCP and the Complex I inhibitor rotenone plus the complex III inhibitor antimycin A were sequentially injected to evaluate basal respiration, ATP production and maximal respiration of primary neuronal cells.

Interestingly, we found that *α*-syn null neurons exhibited a significant decrease in basal respiration when compared to that of wt control cells. Maximal respiration (i.e., mitochondrial energetic reserve capability) and ATP production were also reduced in the cortical neurons lacking *α*-syn (Figure 1(b)). These findings support that the absence of *α*-syn compromises the OCR both in basal conditions and in response to FCCP, which, by stimulating the respiratory chain to run at maximum capacity, allows estimating the energetic reserve capability of the cell.

By analyzing OCR, we also found that the *α*-syn null neurons, which were subjected to 1 h rotenone pretreatment, exhibited a significant decrease in basal respiration, maximal respiration and ATP production after the 24 h washout, when compared to untreated *α*-syn null neurons. This finding is in line with evidence supporting that *α*-syn-deficient mice are more sensitive to rotenone and show a more marked degeneration of dopaminergic neurons upon exposure to this neurotoxin (Dauer et al., PNAS, 2002). Conversely, the OCR profile of wt neurons was not affected by 1 h rotenone pretreatment, supporting that these cells displayed a better resilience within our experimental paradigm. This observation supports that in *α*-syn null neurons, the functional activity of mitochondria resulted in more vulnerability to the 1 h Complex I inhibition achieved by rotenone pretreatment.

We examined the ECAR profile, an indirect but reliable index of cellular glycolytic rate [21], in basal conditions and in response to mitochondrial respiratory chain inhibitors in wt and *α*-syn null neurons (Figure 1(a)). We found that the absence of *α*-syn reduced the basal and oligomycin-stimulated ECAR profile when compared to that of wt neurons, regardless of rotenone pretreatment.

While rotenone is known to inhibit neuronal OCR and enhance ECAR when measured during its infusion [22], we found that 1 h rotenone pretreatment *per se* did not affect ECAR and OCR in wt neuronal cells. This could be explained by the recovery of neuronal bioenergetic capacity during the 24 h washout period. The combined evaluation of the OCR and ECAR parameters in basal conditions, as shown in the energy map (Figure 1(a)), indicated that wt neurons have higher bioenergetics capacity, whereas *α*-syn null neurons have a more quiescent metabolic phenotype.

Finally, we analyzed mitochondrial OCR also in mitochondria purified from liver, cortices, and midbrain of adult wt and *α*-syn null mice, but we did not detect any difference in respiration or ATP production (Figures 1(c) and 1(d)). This may possibly be ascribed to the fact that *α*-syn is enriched in neurons. Therefore, *α*-syn absence would not affect mitochondrial respiration in peripheral tissues and its effect may be hardly detectable in brain mitochondrial preparations due to the presence of mitochondria deriving from glial cells, which do not normally express the protein [23–25]. Contrariwise, the expression of *α*-syn in neurons is elevated, especially at synaptic sites [26], where mitochondria are also abundant [27]. This supports that *α*-syn may affect mitochondrial homeostasis and impinge on respiration exclusively in neuronal cells.

Collectively, these findings support that *α*-syn null neurons exhibit a reduced respiration and are also less energetic, thus supporting that *α*-syn plays a relevant role in orchestrating mitochondrial functions and energy production.

**Figure 2:**
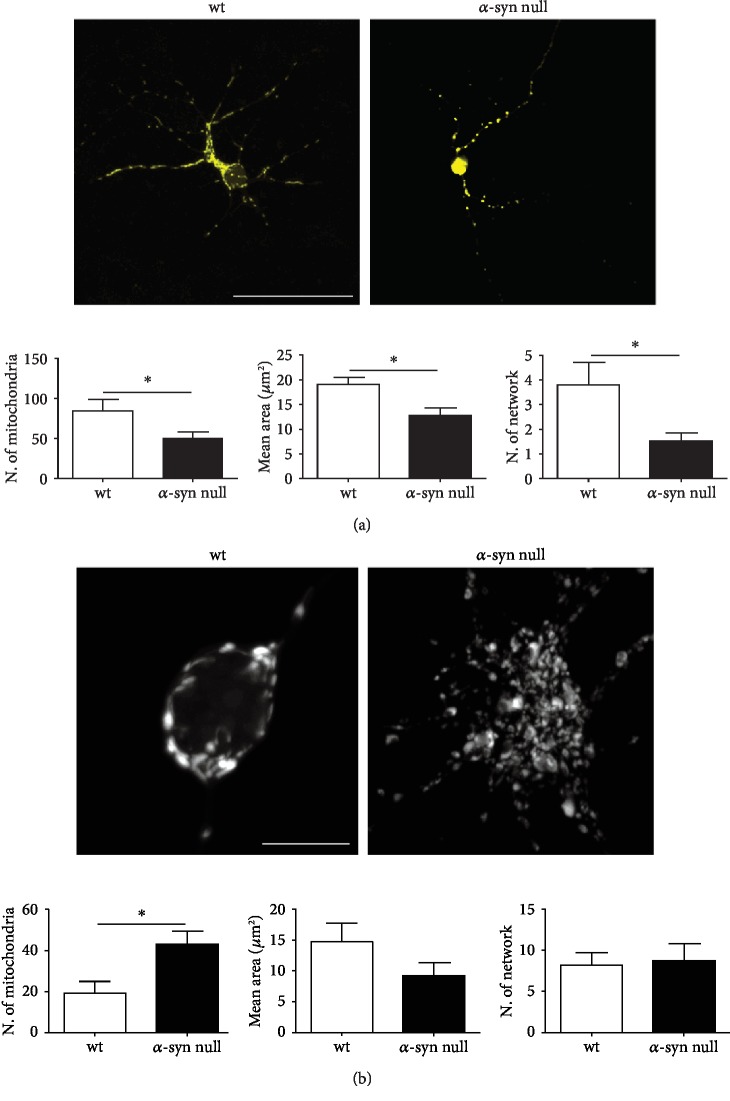
Mitochondrial morphology in mito-YFP transfected primary cortical neurons of wt and *α*-syn null mice. (a) Representative images of maximum intensity projections of primary cortical neurons from wt and *α*-syn null mice transfected with mito-YFP construct at 8 DIV. The morphological analysis showed decreased number of mitochondria exhibiting also reduction in the mean area and in the number of interconnections in those of *α*-syn null mice when compared to wt neurons. (b) Maximum intensity projection of Airyscan superresolution microscopy showed an increased number of mitochondria in the cell body of *α*-syn null neurons which did not exhibit reductions in the mean area or in their interconnections when compared to those of wt mice. ^∗^*P* < 0.05, unpaired two-tailed *t*-test. Data are presented as mean ± SEM (*n* = 30). Scale bar: *a* = 50 *μ*m and *b* = 10 *μ*m.

It has been found that, in the presence of *α*-syn aggregates, neurons show fragmented mitochondria [28, 29]. Considering that *α*-syn aggregation may mimic a loss of function of the protein [30], we analyzed the morphology of mitochondria in the MAP-2-immunolabelled cortical neurons from wt and *α*-syn null mice which had been transfected with mito-YFP construct (Supplementary [Supplementary-material supplementary-material-1]) to discriminate mitochondria within the cell soma and dendrites (Figure 2). These areas were separately examined, as dendrites were acquired by standard confocal microscopy, while cell somas were acquired by Airyscan superresolution microscopy to allow a better visualization of mitochondrial morphology.

By analyzing the dendrites of *α*-syn null neurons, we found a decrease in the number of mitochondria, which also exhibited a reduction in the mean area when compared to those of wt neurons (Figure 2(a)). This is in line with previous studies showing increased mitochondrial fragmentation after *α*-syn silencing in HeLa cells [31]. Moreover, the number of interconnections between mitochondria, analyzed by using the MiNA toolset, was also significantly decreased in *α*-syn null neurons. The reduction of mitochondrial contacts and mitochondrial mean area may be indicative of mitochondrial fission/reduced fusion [32, 33] occurring in the absence of *α*-syn.

Interestingly, when we analyzed mitochondria number and morphology in the cell bodies, we found that *α*-syn null neurons exhibited an increased number of mitochondria, whose mean area only showed a trend toward size reduction, which however did not show significant results with respect to wt cells (Figure 2(b)). Moreover, the absence of *α*-syn did not result in a decrease of mitochondria interconnections in cell bodies. These findings suggest that *α*-syn null neurons may present an impairment of mitochondrial transport along processes, which may show mitochondrial fission as a consequence of this process [34, 35].

Mitochondrial fragmentation may be associated with changes in the expression of fusion and fission proteins [35]. While mitochondrial fusion is considered a prosurvival mechanism [36], fission/fragmentation is often an index of mitochondria damage.

**Figure 3:**
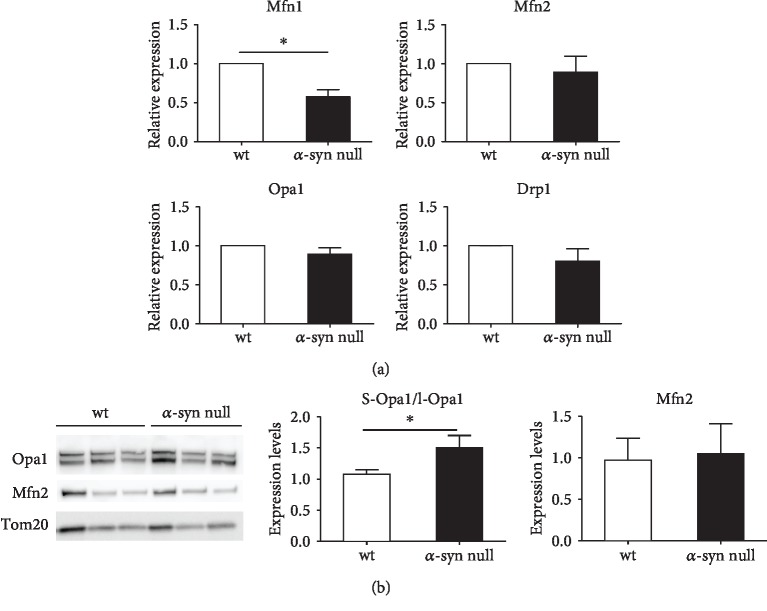
Expression levels of mitochondrial proteins involved in shape modification. (a) Relative expression of Mfn1, Mfn2, Opa1, and Drp1 was evaluated by using real-time PCR on primary cortical neuron extracts of wt and *α*-syn null mice. Note the statistically significant reduction of Mfn1 in the primary cortical neurons from *α*-syn null mice. (b) Western blot analysis revealed increased ratio between the short and long forms of Opa1 in the absence of *α*-syn which did not exhibit alterations in the levels of Mfn2. ^∗^*P* < 0.05, unpaired two-tailed *t*-test. Data are presented as mean ± SEM (*n* = 8).

We thus analyzed the expression levels of genes involved in mitochondrial shape modifications: Mfn1, Mfn2, Opa1, and Drp1 (Figure 3(a)). In the cortical neurons of *α*-syn null mice, we observed decreased levels of Mfn1, which is involved in the fusion of the outer mitochondrial membrane, but no changes in the expression of the fission protein Drp1 or in Opa1 and Mfn2. This finding supports that, by modulating Mfn1 expression, *α*-syn may ensure mitochondrial fusion but does not seem to affect proteins controlling the fission, stability, or formation of these organelles. This is in line with evidence indicating that *α*-syn aggregation inhibits mitochondrial fusion through a Drp1-independent pathway [37].

The absence of Mfn2 changes was confirmed by western blot analysis (Figure 3(b)), which showed an interesting increase in the ratio between the short and long forms of Opa1, which is crucially involved in control of mitochondrial morphology. This observation supports that the absence of *α*-syn can promote mitochondrial fission also by reducing long Opa1, which is relevant for conferring fusion competences [38]. On the other hand, an increased short Opa1 generation can promote fragmentation [39].

**Figure 4:**
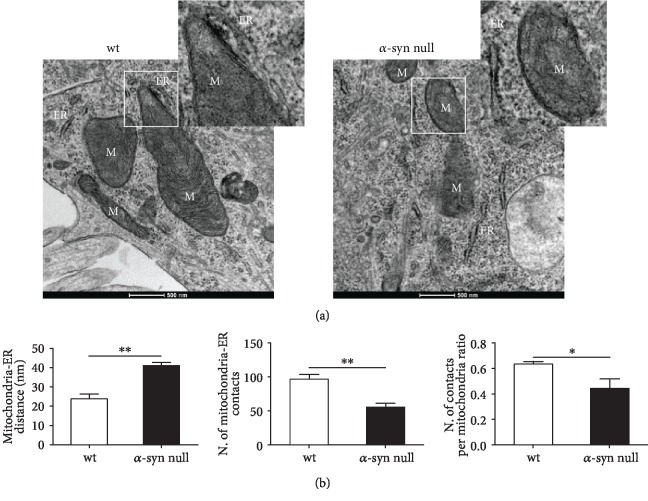
TEM-based morphological analysis of mitochondria-ER interaction of primary cortical neurons of wt and *α*-syn null mice. (a) Representative images of TEM showing the mitochondria-ER interactions in primary cortical neurons of wt and *α*-syn null mice. ER = endoplasmic reticulum; M = mitochondria. Scale bar: 500 nm. (b) The image analysis showed an increased distance between mitochondria and ER, a decreased number of MERCs, and a decreased ratio in the number of contacts per mitochondria in *α*-syn null neurons when compared to wt neurons. ^∗^*P* < 0.05 and ^∗∗^*P* < 0.01, unpaired two-tailed *t*-test. Data are presented as mean ± SEM (*n* = 5).

Since the presence of wt *α*-syn was previously observed in MAM [4] and mitofusins are known to be involved in mediating MERCs [40–42], we also analyzed mitochondria-ER interactions by TEM (Figure 4). Indeed, Mfn2 is known to have a key role in the mitochondria-ER tethering but is also known to form homo- or heterodimerization with Mfn1. By localizing to MAM [4], *α*-syn absence could modify Mfn2 localization, thus consequently influencing mitochondria-ER interaction. The results of this part of the study showed a decreased number of MERCs in the *α*-syn null neurons when compared to that of wt neurons that still significantly decreased when normalized on the number of mitochondria. In parallel, we observed an increased distance between mitochondria and the ER. MERCs are relevant for ensuring mitochondrial biogenesis, dynamics, and inheritance and Ca^2+^ release from ER at MERC controls mitochondrial function, division, and regulation of apoptosis [43]. Our data confirm and extend previous studies showing alterations in MAM structure due to *α*-syn mutations [4] and the disruption of ER-mitochondrial contacts caused by *α*-syn overexpression [44], which may coincide with a loss of *α*-syn function. Further research is required to assess whether the reduction of MERCs and increased mitochondria-ER distance observed in the *α*-syn null mice might play a causal role in the altered mitochondrial function and dynamics.

## 4. Conclusions

Collectively, the results of this study support that *α*-syn plays a physiological and essential role in the control of mitochondrial respiration capacity and homeostasis.

Alpha-synuclein aggregation and mitochondrial defects are believed to be central in the pathogenesis of neurodegeneration in PD [3, 10, 45, 46]. This is clearly reinforced by the fact that mutations of *α*-syn or mitochondria-associated genes can cause the onset of familial early-onset parkinsonism [47, 48]. Interestingly, recent evidence pointed out that *α*-syn localizes in and affects MAM function [4, 16, 49] and that the N-terminus of *α*-syn, a region exhibiting high affinity for biological membranes [50], can control mitochondrial membrane permeability [51]. Moreover, *α*-syn can interact with Complex I modulating its activity [52], while *α*-syn overexpression induces mitochondrial fission by interacting with mitochondrial membranes [5]. The *α*-syn-mediated control of mitochondrial homeostasis, which is not altered by the A30P variant, is selectively disrupted by the A53T mutation [45]. Consistently, A53T transgenic mice show a marked reduction of the Na^+^-Ca^2+^ exchanger 3 (NCX3) accompanied by mitochondrial Ca^2+^ overload, events which have been proposed to be central for neurodegeneration of dopaminergic neurons in this mouse line [53]. These studies, strongly supporting a role for *α*-syn in mitochondrial homeostasis, fail to provide information on the physiological role of *α*-syn on morpho-functional aspects of mitochondrial biology. In line with the Complex I deficit previously described by Devi and colleagues [52], electron transport chain impairment, with no changes in mitochondrial number, has been demonstrated in mice lacking *α*-syn [54]. Nevertheless, a complete characterization of the physiological effects of *α*-syn on mitochondrial morphology and activity in pure neuronal preparations have never been investigated before, with the exception of a single study that however failed to detect differences in mitochondrial bioenergetics between wt and *α*-syn ko mice [55].

Remarkably, our results are partially in line with those described by Pathak et al. as when we analyzed mitochondria purified by cortical tissues, we also failed to detect functional differences. Differently from their findings on primary hippocampal neurons prepared from *α*-syn ko pups, when we analyzed primary cortical neurons from *α*-syn null mouse embryos, we found that they exhibited significant reduction in basal and maximal respirations as well as ATP production when compared to those from wt mouse embryos. Moreover, *α*-syn null neurons resulted in more vulnerability to rotenone treatment, supporting that the effect of this toxin is influenced by the presence of *α*-syn. The functional impairments were accompanied by marked reduction of MERCs as well as by mitochondrial morphology alterations supportive of the occurrence of fragmentations within dendrites and reduction of mitochondria transport. Remarkably, the expression of *α*-syn can vary between diverse brain areas and different neuronal populations [56], thus supporting that the protein may differentially impinge on mitochondrial functions in hippocampal or cortical neurons. Therefore, the discrepancies between our findings and those described by Pathak et al. can be the result of different factors: (a) we analyzed different neuronal subpopulations (whole cortices vs. hippocampi); (b) these were prepared at different time points (embryos vs. pups); and (c) we used different strains and experimental models (C57BL/6J *α*-syn null vs. C57BL/6N *α*-syn ko mice).

Notably, our results sound in agreement with multiple evidence supporting that *α*-syn overexpression and mutations can influence mitochondrial homeostasis and fragmentation as well as ER-mitochondrial interaction [4, 5, 46, 52]. However, the physiological effect *α*-syn on mitochondria function, morphology, and interaction with ER had been only partially addressed up to date [4, 5, 37, 57]. Our results support that *α*-syn can physiologically affect the mitochondrial functional profile, preserves mitochondrial fusion and transport, and contributes to ensure MERCs in neuronal cells. When considering the central role that *α*-syn aggregation plays in PD, these observations support that the absence of the protein in C57BL/6JOlaHsd null neurons may mimic the effects derived from *α*-syn insoluble inclusion formation, which, by sequestering the protein, may reduce its functional profile [3, 30, 46]. This process may severely alter mitochondrial and mitochondria/ER dynamics, thus promoting neuronal damage and degeneration.

## Data Availability

The datasets used and/or analyzed during the current study are available from the corresponding author on reasonable request.

## Supplementary Materials

Supplementary Materials Supplementary Figure 1: representative photomicrographs showing MAP-2-labelled neurons transfected with mito-YFP construct of wt and *α*-syn null mice. Scale bar: 20 *μ*m.
