# Supplementary figures and images for: Alpha-Synuclein Preserves Mitochondrial Fusion and Function in Neuronal Cells
Source: Oxid Med Cell Longev. 2019 Nov 23;2019:4246350. doi: 10.1155/2019/4246350 (PMC6907050; doi:10.1155/2019/4246350)

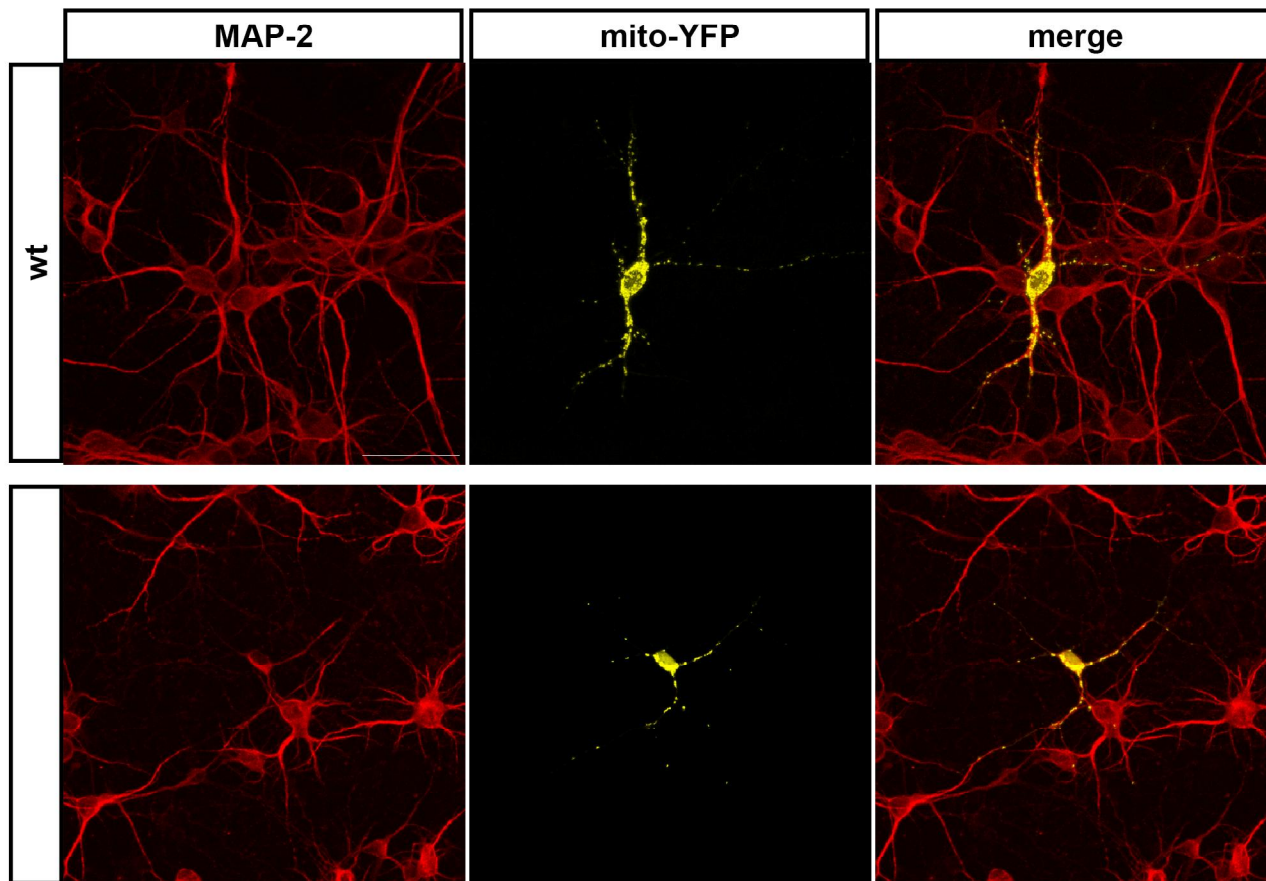

Supplement: Supplementary Materials — Supplementary Figure 1: representative photomicrographs showing MAP-2-labelled neurons transfected with mito-YFP construct of wt and α-syn null mice. Scale bar: 20 μm. [file 4246350.f1.pdf]
